# Identification of high-risk extended-spectrum β-lactamase-producing *Escherichia coli* clones harboring *tet*(X4) along the pork supply chain in central Thailand

**DOI:** 10.1371/journal.pone.0355459

**Published:** 2026-08-13

**Authors:** Variya Nemidkanam, Ramzan Ahmed, Nuntaree Chaichanawongsaroj

**Affiliations:** Department of Transfusion Medicine and Clinical Microbiology, Faculty of Allied Health Sciences, Center of Excellence for Innovative Diagnosis of Antimicrobial Resistance, Chulalongkorn University, Bangkok, Thailand; Zhejiang University, CHINA

## Abstract

The global dissemination of antimicrobial-resistant (AMR) bacteria along the pork supply chain is a critical concern, as pigs are significant reservoirs for multidrug-resistant (MDR) *Escherichia coli*, a WHO-listed priority pathogen. This study investigated the genomic characteristics of extended-spectrum β-lactamase (ESBL)-producing *E. coli* across the pork production chain in Central Thailand, specifically focusing on the emergence of the last-resort tigecycline resistance gene, *tet*(X4). ESBL-producing *E. coli* isolates from a slaughterhouse pig cecum (n = 14) and retail market pork (n = 26) in central Thailand were collected. Whole-genome sequencing (WGS) was employed to analyze the resistome, virulome, and phylogenomic relationships. Analysis included non-metric multidimensional scaling (NMDS) of resistance and virulence gene determinants, single-nucleotide polymorphism (SNP) phylogeny, and core genome MLST (cgMLST). Resistome, virulome, and NMDS analysis demonstrated a shared clustering of antimicrobial resistance genes (ARGs) and virulence factors (VF) genes between cecum and pork isolates. Most isolates possessed extraintestinal pathogenic *E. coli* (ExPEC)-associated VF genes, underscoring the widespread pathogenic risk. *tet*(X4) was predominantly found in the dominant ST48 clone and these *tet*(X4) adjacent to IS*CR2*, providing evidence of horizontal gene transfer (HGT). cgMLST further demonstrated that these *tet*(X4) isolates are genetically related to global human, animal, and environmental strains. This study provides the first genomic evidence of *tet*(X4) circulation in ESBL-producing *E. coli* population across the slaughterhouse-to-retail continuum in central Thailand. Our findings suggest that the dissemination of *tet*(X4) in the pork supply chain is primarily via HGT. The implementation of firm food safety policies and enhanced hygiene practices is urgently required to inhibit the transmission of these high-risk strains to consumers.

## Introduction

The global spread of antimicrobial resistance (AMR) within the One Health cycle is a critical public health threat. The Codex Guidelines for Risk Analysis of Foodborne AMR specifically define antimicrobial-resistant microorganisms as significant microbiological food safety hazards [1]. The European Union surveillance program, which monitors AMR in the major meat-producing species, utilizes *Escherichia coli* and enterococci as primary indicators for assessing overall food safety [2].

Pigs represent a crucial reservoir for the emergence and circulation of high-priority multidrug-resistant bacteria (MDR). An escalating global demand for pork further highlights this public health concern. Pork consumption accounts for approximately 34% of global meat intake, with demand projected to rise from 131 million tons per year to 131.5 million tons per year by 2031 [3]. Asia produces 60% of the world’s pork; in Thailand, particularly high recorded levels of antimicrobial use in livestock create intense selective pressure for the emergence of novel resistance determinants. While exported pork is typically raised under high-standard farming and food safety conditions [4], continuous surveillance of products for domestic consumption remains essential to prevent AMR dissemination. AMR contamination can occur throughout the supply chain, starting from slaughter transportation to retail sales and preparation. This risk is supported by several studies confirming the existence of MDR in a variety of meat samples collected from retail markets [5]. Critical antimicrobial-resistant genes (ARGs) in *E. coli*, such as extended-spectrum β-lactamases (ESBLs), *mcr-1*, and *tet* genes, have been recovered from many meat products [6]. Among the diverse lineages of *E. coli* circulating worldwide, Clonal Complex 10 (CC10), containing ST10, ST44, ST48, ST167, and ST617, has emerged as globally disseminated vehicles for critical antimicrobial resistance (AMR) traits. These lineages are recognized as the most common high-risk clone due to their alignment with essential epidemiological criteria, which include (a) widespread dissemination across multiple hosts or environmental settings; (b) a high capacity for transmission, persistence, and pathogenicity; and (c) the carriage of multiple antimicrobial characteristics, especially the WHO critical priority of the antimicrobial resistance list [7,8].

Tigecycline is one of the last resort antibiotics for treating MDR infections. However, the clinical utility of this critical agent is increasingly threatened by the emergence of plasmid-mediated resistance genes, most notably *tet*(X4). It was first identified in *E. coli* from swine in 2019 [9], *tet*(X4) encodes a flavin-dependent monooxygenase that enzymatically inactivates all generations of tetracyclines, including tigecycline [10]. Enterobacterales harboring *tet*(X4) were detected from the food production chain and vegetables [11–13]. The emergence of tigecycline resistance in animals might be associated with the long-term exposure to and extensive use of tetracyclines and phenicols, particularly in swine farms worldwide [14–16]. The rapid dissemination of *tet*(X4) in livestock is particularly concerning as it is possible to co-occur with other prevalence genes such as ESBLs. This co-carriage can create “superbugs” that are resistant to nearly all standard treatment options, severely limiting therapeutic interventions in both veterinary and human medicine. The high mobility and rapid spread of the *tet*(X4) gene are frequently driven by IS*CR2*-mediated transposition. IS*CR2* facilitates the movement of adjacent resistance determinants through rolling-circle transposition, allowing the *tet*(X4) cassette to jump between diverse plasmid backbones and chromosomal locations [17]. This enables the transmission of *tet*(X4) across different bacterial hosts and environments.

While the emergence of ESBL-producing bacteria and the *tet*(X4) gene has been documented across Asia, their genomic dissemination within Thailand’s pork production chain remains unexplored. This study aims to characterize the resistome, virulome, mobilome, sequence types, and perform phylogenetic analysis to reveal the complex evolutionary trajectories of ESBL-producing isolates from pork and cecum samples using Whole Genome Sequencing (WGS). Furthermore, we investigated the genetic context driving the mobilization and spread of *tet*(X4) in these isolates.

## Materials and methods

### Sample collection, bacterial culture, and antimicrobial susceptibility testing (AST)

*E. coli* isolates used in this study were derived from pig cecum samples (code: SP) and retail pork meat samples (code: PE) originally collected during a previous study [18]. Briefly, a comprehensive epidemiological survey of ESBLs in retail markets was generalized by sampling 100 pork shops distributed across 50 districts in the Bangkok Metropolitan Region, representing the consumer-level end of the supply chain. One slaughterhouse was selected from Suphan Buri, Thailand, which is one of the major suppliers of pork to numerous retail markets in Bangkok. One hundred pork (250 g/sample) and 50 pig cecum samples were collected and transported on ice. ESBL*-*producing *E. coli* was isolated on MacConkey agar supplemented with 1 g/L cefotaxime and confirmed using the combination disk method according to the Clinical and Laboratory Standards Institute (CLSI) guideline [19]. The ESBL genes were characterized by PCR sequencing.

The purposive sampling was then designed to select representatives of each ESBL variant identified in retail market pork across 19 districts and cecum isolates from a slaughterhouse. A subset of ESBL variants that balanced across the listing source, phenotype, and ESBL genotypes (S1 and S2 Tables) was selected as a model to explore the molecular transmission from a slaughterhouse to markets. These representative ESBL isolates were subcultured on MacConkey agar (Oxoid, UK), incubated at 37°C, and subsequently subjected to WGS analysis.

### Whole genome sequencing (WGS) and bioinformatic processing

**Fig 1:**
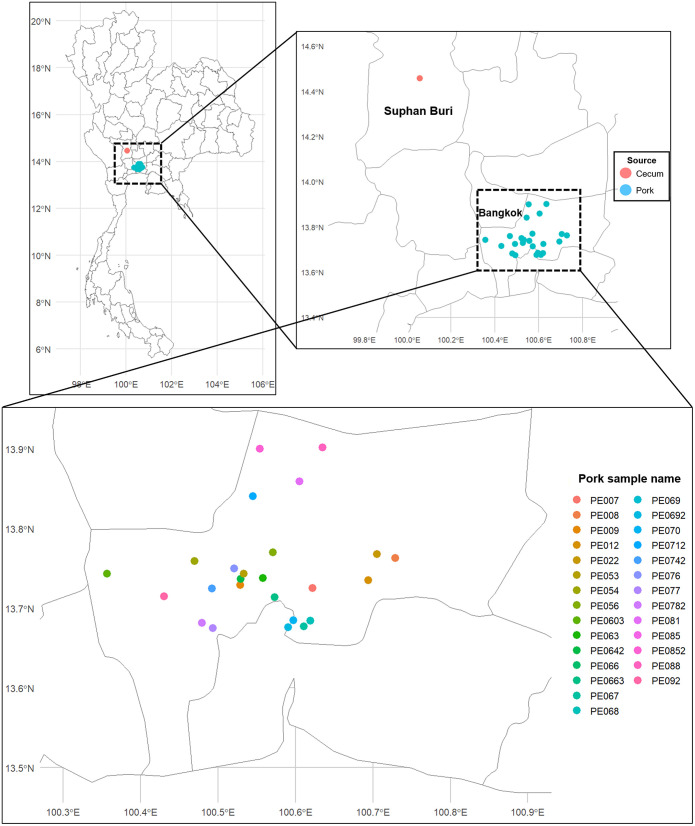
Geographic distribution of sampling locations in the central Thailand area. The map indicates the collection location for the representative *E. coli* isolates used in this study. Cecum isolates (n = 14, code: SP) were obtained from a wholesale slaughterhouse located in Suphan Buri, Thailand. Pork isolates (n = 26, code: PE) were collected from retail markets distributed across Bangkok’s metropolitan area. The base map and administrative boundaries were generated using public domain vector data sourced from Natural Earth (https://www.naturalearthdata.com/).

A targeted molecular sampling framework was deployed to select representative isolates for WGS. This sub-selection was based on previous β-lactamase gene profiles performed by genotyping (*bla*_CTX-M_, *bla*_TEM_, *bla*_OXA_, *bla*_SHV_) to ensure that all circulating variants were adequately represented (S2 Table). Fourteen isolates from cecum samples were purposively selected from the 48 presumptive ESBL-producing *E. coli* that possessed 9 distinct β-lactamase gene profile combinations. Twenty-six (n = 26) isolates from retail pork meat samples were representatively selected from the 92 presumptive ESBL-producing *E. coli* that possessed 27 distinct β-lactamase gene profile combinations [18]. To ensure adequate coverage across Bangkok’s metropolitan area, the geographical locations of selected isolates were mapped and visualized using the ggplot package in RStudio (Fig 1).

The selected *E. coli* isolates were subjected to DNA extraction using the ZymoBIOMICS DNA Extraction Kit (Zymo Research, USA) following the manufacturer’s protocol. WGS data were obtained through the short-read Illumina MiSeq platform, provided by MicrobesNG (Birmingham, UK), providing 2 x 250 bp paired-end reads with a standard factory target coverage depth of ≥ 30X per isolate. Raw reads were quality-trimmed using Trimmomatic v0.30 with a 4-bp sliding window quality threshold of Q15, discarding reads shorter than 36 bp. Assembly was performed using SPAdes v3.7, and annotation of contigs was carried out with Prokka 1.14.3. Reads were aligned to the reference genome with BWA-MEM v0.7.17 and processed further using SAMtools v1.9. Genomic quality control (QC) thresholds were applied across all isolates. Assemblies were only accepted for downstream molecular and epidemiological characterization if they fulfilled the following standardized criteria: an average sequencing depth of coverage ≥ 30X, a total finalized genome assembly size within the typical expected range for *E. coli* (4.5 to 5.5 Mb), and an N50 value greater than 50,000 bp, indicating strong assembly contiguity. Detailed isolate-specific metadata, NCBI BioProject/SRA, and genomic quality control metrics (including individual mean coverage depths, total assembly sizes, %GC, and N50 value) for all genomes are comprehensively detailed in S2 Table.

### Resistome and Virulome Profiling

All isolates were analyzed to identify acquired antimicrobial resistance genes (ARGs) and chromosomal mutations associated with fluoroquinolone resistance using ResFinder 4.6.0 (http://genepi.food.dtu.dk/resfinder). Virulence factor (VF) genes were identified using VirulenceFinder 2.0 (https://cge.food.dtu.dk/services/VirulenceFinder).

### Bacterial typing and classification

Multilocus Sequence Typing (MLST) was performed with MLST 2.0 (https://cge.food.dtu.dk/services/MLST/) to assign sequence types (STs) based on the Achtman 7-locus scheme. Ribosomal sequence typing (rMLST) was conducted by querying assemblies against the PubMLST database (https://pubmlst.org/). Serotypes (O and H antigens) were identified using SerotypeFinder 2.0 (https://cge.food.dtu.dk/services/SerotypeFinder/). Isolates were classified into seven phylogroups (A, B1, B2, C, D, E, and F) using In Silico Clermont Phylotyper [20]. Pathotyping was conducted based on the presence of candidate virulence factor genes. Isolates were assigned to the main extraintestinal pathogenic *E. coli* (ExPEC) pathotype if they could be classified into any individual ExPEC sub-pathotype (UPEC, APEC, and NMEC) by exhibiting the presence of two or more candidate virulence genes within that specific sub-pathotype [21].

### Phylogenetic tree analysis

Single-nucleotide polymorphism (SNP) analysis was conducted using the Reference Sequence Alignment-based Phylogeny builder (REALPHY) v1.12. Phylogenetic trees were visualized and annotated with the Interactive Tree of Life (iTOL). *E. coli* K-12 (GenBank accession: NC_000913.3) was used as the reference genome. To visualize the clonal relationships and SNP distances among the isolates, a clustered heat map was generated using the ggplot package in RStudio.

### MGEs co-contig with ARGs and VF genes

Plasmid typing (Inc typing) and other MGE determinants were identified from the assembled contigs. Plasmid replicons were determined using PlasmidFinder 2.1 (https://cge.food.dtu.dk/services/PlasmidFinder), while non-plasmid MGEs were identified using MobileElementFinder 1.0.3 (https://cge.food.dtu.dk/services/MobileElementFinder). A co-contig analysis was performed to identify where MGEs and ARGs/VF genes were located on the same assembled contig fragment. Co-occurrence results were visualized using a bubble plot, generated using the ggplot2 package in RStudio.

### ST48 *tet*(X4)-harboring contig-based comparative analysis

Assembled contigs of *E. coli* ST48 harboring *tet*(X4) gene were analyzed to characterize their genetic architecture and identify potential mobile elements. Sequence similarity searches were conducted using Microbial Nucleotide BLAST against *E. coli* (taxid:562) complete genome database. Reference plasmid sequences exhibiting the highest identity and query coverage were retrieved for comparative analysis. The genetic environment of *tet*(X4) was aligned and visualized using Easy Fig 2.2.3 to illustrate structural conservation across isolates.

### Core genome MLST (cgMLST) analysis of *tet*(X4)-harboring isolates

cgMLST was performed using the EnteroBase platform (https://enterobase.warwick.ac.uk) to assess genetic relatedness and retrieve closely related strains from the global database. Isolates were characterized by using the *E. coli* cgMLST scheme (comprising 2,513 core loci). Genetic clusters were defined using the Hierarchical Clustering of cgMLST (HierCC) at the HC100 level, which identifies groups of isolates with pairwise differences of ≤100 alleles. The phylogenetic relationship was visualized as a minimum spanning tree using GrapeTree (MSTree V2 algorithm) with associated metadata.

### Statistical analysis

To visualize the similarities in the overall ARGs and VF gene profiles, a non-linear multidimensional scaling (NMDS) plot was generated. This plot was based on the Jaccard distance matrix calculated from the binary (presence/absence) profiles of ARGs and VF gene profiles. To statistically evaluate differences in these profiles across different sample sources, a Permutational Multivariate Analysis of Variance (PERMANOVA) was performed with 999 permutations using the adonis2 function. Furthermore, to distinguish whether observed variations were driven by true structural shifts between groups or differences in within-group heterogeneity, a test for homogeneity of multivariate dispersions (beta-dispersion) was conducted using the betadisper function. All multivariate statistical analyses and visualizations were executed using the vegan and ggplot2 packages in RStudio.

## Results

### Resistome and fluoroquinolone resistance-associated chromosomal mutations

**Fig 2:**
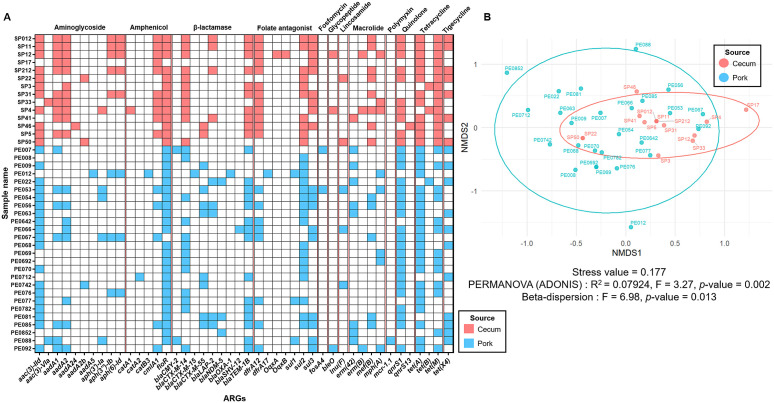
Resistome of *E. coli* isolates from pig cecum and retail pork meat samples. (A) Distribution of ARGs across all isolates. (B) An NMDS plot illustrating the similarities of ARG profiles among isolates from the cecum and pork isolates.

The resistome analysis of the plasmid-associated ARGs demonstrated a diverse array of 45 ARGs belonging to 12 classes of antimicrobial agents in all isolates (Fig 2A). ARGs responsible for resistance to amphenicol, tetracycline, quinolone, and aminoglycoside, including *floR, tet*(A)*, qnrS1,* and *aac(3)-IId*, were commonly found (>70% prevalence) in both cecum and pork isolates.

Pork isolates showed a higher diversity of ARGs (40 ARGs) than cecum isolates (33 ARGs). Furthermore, 12 ARGs that were exclusively found in pork isolates included critical ARGs, such as *bla*_OXA-1_ (1/26 isolates, 3.84%) and *bla*_NDM-5_ (3/26 isolates, 11.54%) for carbapenem resistance and *mcr-1.1* (1/26 isolate, 3.84%) for colistin resistance. Additionally, six of point mutations on *gyrA* and *parC/E* associated with quinolone resistance were also mainly found in pork samples, with *gyrA* p.S83L mutation observed in all pork isolates (26/26 isolates, 100%) (S1 Fig). Interestingly, the *tet*(X4), which confers tigecycline resistance, was frequently found in both cecum (7/14 isolates, 50%) and pork isolates (11/26 isolates, 42.30%). The NMDS plot demonstrated that the overall ARG profiles of isolates from the two sources overlapped, suggesting potential shared resistant mechanisms and transmission pathways (Fig 2B). However, they exhibited a minor but statistically significant difference in their ARG profiles via PERMANOVA(ADONIS) (R^2^ = 0.07924, F = 3.27, *p*-value = 0.002). While the small R^2^ value confirms that only 7.92% of the total ARG is distinctly explained by the sample source. This aligns with the visual overlap and indicates a highly shared core resistome across the supply chain. Additionally, the beta-dispersion analysis was significant (*p*-value = 0.013), indicating that the groups also differ in their internal variation, with retail pork isolates exhibiting a more scattered and heterogeneous distribution than the tighter cluster of pig cecum isolates.

Furthermore, all isolates were previously classified as ESBL producers. This mechanism is supported by the variety of β-lactamase genes, with 67.5% (27/40 isolates) of all isolates harboring more than one β-lactamase gene, including *bla*_CTX-M-55_, *bla*_LAP-2_, *bla*_NDM-5_, *bla*_CMY-2_, *bla*_CTX-M-15_*, bla*_OXA-1_*,* and *bla*_SHV-12_.

### Genotypic virulence characterization

**Fig 3:**
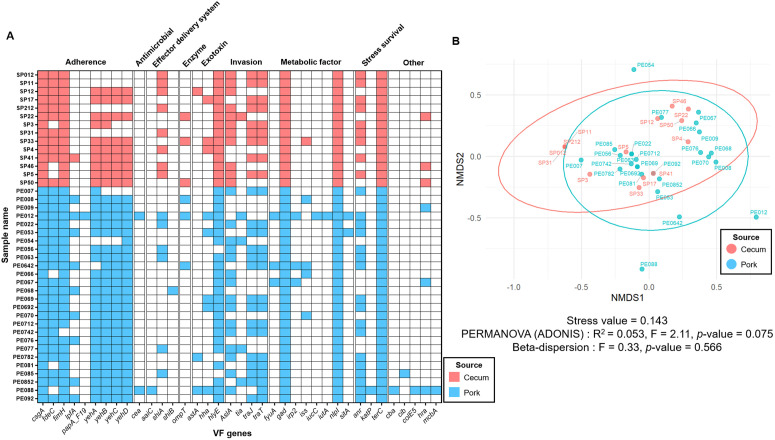
VF gene profiling of *E. coli* isolates from pig cecum and retail pork meat samples. (A) The distribution of VF genes among isolates categorized by their function. (B) An NMDS plot visualizing the overall VF gene profiles of cecum and pork isolates.

The analysis identified 37 VF genes involved in several main pathogenesis pathways, including adherence (9 genes), antimicrobial (1 gene), effector delivery system (3 genes), enzyme (1 genes), exotoxin (3 genes), invasion (4 genes), metabolic factor (8 genes), stress survival (3 genes), and others with unknown functions (5 genes) (Fig 3A). While cecum isolates shared all identified VF genes with pork isolates, uniquely possessed 14 VF genes. An adherence gene (*csgA*), a stress survival gene (*terC*), and two metabolic factor genes (*gad* and *nlpI*) were consistently found in all isolates (40/40 isolates, 100%). Additionally, other adherence genes (*fdeC*, *fimH,* and *yeh*) and exotoxin gene (*hlyE*) were also predominantly found across all isolates. Some VF genes were exclusively found in *E. coli* isolated from pork samples, such as *cea*, *cba, fyuA*, and *irp2*. The NMDS plot demonstrated that the VF gene profiles of isolates from the two sources exhibited a high degree of overlap, suggesting a closely shared virulence potential (Fig 3B). The PERMANOVA (ADONIS) (R^2^ = 0.053, F = 2.11, *p*-value = 0.075) confirmed the visual similarity and internal variation within each group were highly homogeneous (beta-dispersion: *p*-value = 0.566).

### MGEs and co-localization of ARGs/VF genes

**Fig 4:**
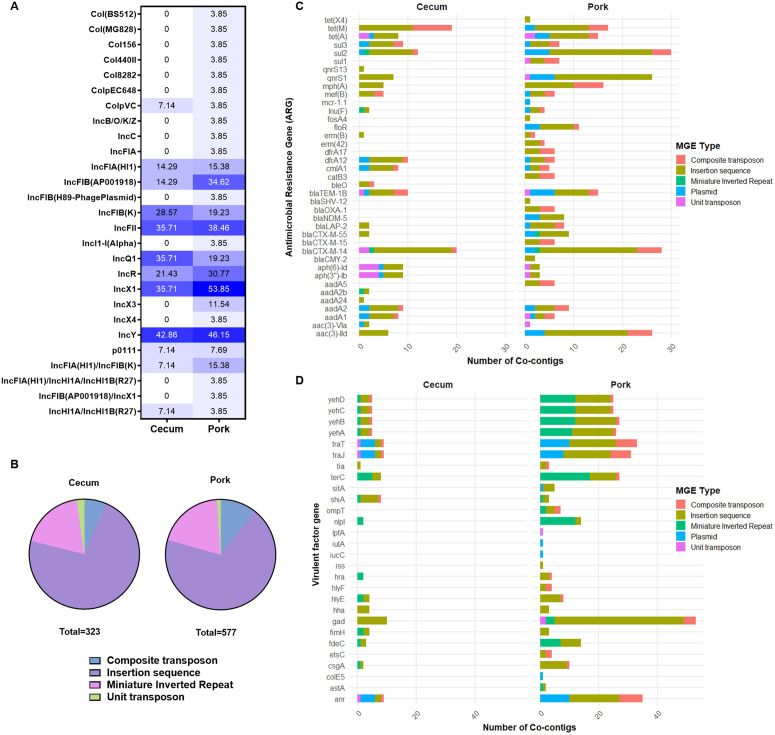
MGEs distribution and co-contig analysis. (A) Prevalence of Inc types identified in both cecum and pork isolates. (B) Frequency of non-Inc MGEs types and their distribution in both cecum and pork isolates. (C) Number of MGEs-ARGs co-contigs categorized by MGE types. (D) Number of VF genes-ARGs co-contigs categorized by MGE types.

Plasmid typing identified 27 distinct incompatibility (Inc) types across all isolates. Pork isolates demonstrated higher diversity compared to cecum isolates, with 12 Inc types shared between the two sources (Fig 4A). Notably, multi-replicon contigs, which are characterized by the presence of distinct Inc types on a single assembled contig, were observed predominantly in pork isolates. The most abundant Inc types were IncX1 in pork isolates and IncY in cecum isolates. Furthermore, the prevalence of IncY, IncFII, IncFIA, and p0111 was comparably observed in both sources. The insertion sequences (IS) were the most frequent MGEs in isolates from both sources, followed by miniature inverted repeat transposable elements (MITEs) and unit transposons, respectively (Fig 4B).

The co-contig analysis revealed linkage between a total of 39 ARGs with each type of MGEs (Fig 4C). Overall, *bla*_CTX-M-14_ was the most co-contig with MGEs across all isolates. However, source-specific patterns showed *bla*_CTX-M-14_ was predominantly found co-contig with MGEs in cecum isolates, while *qnrS1* was most co-contig in pork isolates.

As plasmids are primary ARGs vehicles, IncX1, IncFII, and IncFIA were identified to be predominant Inc-ARGs co-contigs in all isolates (S3 Table). IS was the most frequent MGEs identified co-contig with ARGs, including IS*102*, IS*26*, IS*Aba1*, IS*Ec9*, IS*Kpn19*, and IS*Kpn26*, were also observed in both sources (S4 Table). These major Inc-ARGs and IS-ARGs co-contigs demonstrate shared genetic determinants circulating between the cecum and pork isolates (S2A Fig). Especially, the occurrence of the IS*26*-ARGs was the most frequently observed determinant between the two sources, particularly associated with crucial β-lactam resistance genes, including *bla*_CTX-M-14_. IS*102*, IS*Ec9*, and IS*Kpn19* were also found co-contig with *bla*_CTX-M-14_ and *bla*_CTX-M-55_.

The VF gene *gad* was the most frequently detected VF gene associated with MGEs, primarily IS elements (Fig 4D). Both IncFII and IS*26* exhibited predominantly co-contig with the set of VF genes (*anr*, *traJ*, and *traT*) in isolates from pork and cecum (S2B Fig).

### Population structure and *tet*(X4) distribution

**Fig 5:**
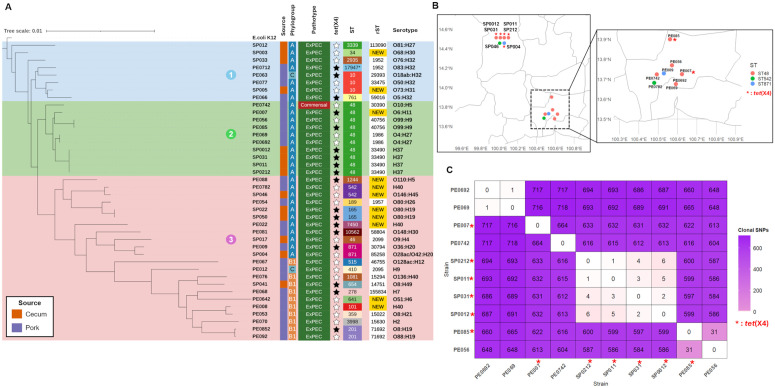
Population structure and clonal relatedness of *E. coli* isolates from pig cecum and retail pork meat samples. (A) SNP-based phylogenetic tree of all *E. coli* isolates. The tree visualizes the specimen type, phylogroup, pathotype, *tet*(X4), STs, rSTs, and serotype of each isolate. The tree was rooted using *E. coil* K-12 as a reference genome, and three distinct clustered clades are numbered on the tree. (B) Geological location of shared STs isolates. Map illustrating the locations of retail pork isolates that share STs with slaughterhouse cecum isolates. The underlying geospatial layers and administrative boundaries are public domain data obtained from Natural Earth (https://www.naturalearthdata.com/). (C) SNP distance matrix of ST48 isolates. Clustered heatmap visualizing the SNP differences among the predominant ST48 isolates from the cecum and pork. Asterisks (*) represent isolates harboring the *tet*(X4) gene, demonstrating the dissemination of high-risk lineages.

Three distinct clades were distributed across both isolates from the cecum and pork (Fig 5A). The isolates were classified into three phylogroups (A, B1, and C). The most predominant was phylogroup A, followed by phylogroup B1, which was mainly found in pork isolates. Phylogroup C was the least prevalent. Based on VF gene markers, ExPEC was identified as a major pathotype across all isolates. The isolates were also classified into 25 different STs. A new ST17947 was discovered from a pork isolate, and new rSTs were found in 11 isolates from both sources. ST48 was the overall predominant ST (10/40 isolates, 25%), and *tet*(X4) was predominantly identified in this clone (6/10 isolates, 60%), especially in cecum isolates. Thirty-two serotypes were found across all isolates. Notably, there was no shared serotype identified between the cecum and pork isolates. Shared serotypes were exclusively found within isolates of the same origin for only some STs. Serotypes H37 and O80:H19 were found to be shared within cecum isolates in ST48 and ST165. Serotypes O4:H27 and O99:H9 were found to be shared within pork isolates in ST48.

Many STs were shared between both cecum and pork isolates, including ST10, ST48, ST542, and ST871 (Fig 5B). However, *tet*(X4)-positive isolates found in both sources were observed exclusively in ST48. Subsequently, these high-risk ST48 isolates were investigated for relatedness due to the potential for cecum-to-pork and spreading within the pork retail market. Therefore, clonal SNP distances of ST48 isolates were analyzed (Fig 5C). Cecum isolates harboring *tet*(X4) showed only 1–6 SNP differences, confirming that they belong to the same clone. Pork isolates from different stalls in the same market (PE069 and PE0692) were closely related, showing only 1 SNP difference. The SNP differences among pork isolates from different markets were more diverse, ranging from 31 SNPs to 718 SNPs. This suggested that the circulation of both closely related clones and distantly related lineages was found across the metropolitan region’s pork supply chain. The SNP distance observed between cecum and pork isolates was found to be relatively high, with 584–694 SNP differences.

### ST48 *tet*(X4) structure

**Fig 6:**
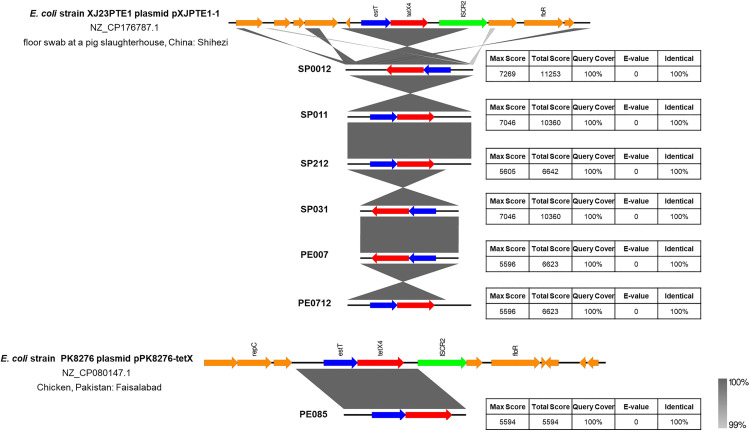
Genetic context of ST48 *tet*(X4)-harboring contig. A visual representation of the genetic context surrounding the *tet*(X4) of each ST48 isolate. Shared genetic context patterns of *tet*(X4) were identified across isolates as indicated. Conserved *estT* flanking genes and IS*CR2* were annotated. Blast result parameters against reference strains were described.

All contigs revealed a highly conserved local genetic architecture across all isolates. The *tet*(X4) was consistently located downstream of the *estT* gene. Furthermore, the mobilization-associated insertion sequence IS*CR2* was identified upstream of *tet*(X4) in several contigs. While IS*CR2* appeared truncated or partial in some sequences, it was likely due to short-read assembly limitations. Comparative BLAST analysis showed that the majority of ST48 *tet*(X4)-harboring contig segments shared 100% sequence identity with *E. coli* strain isolated from a floor swab at a pig slaughterhouse in China (Fig 6). Only one pork isolate exhibited 100% sequence identity to the *E. coli* strain from chicken in Pakistan.

### Comparative genomic analysis of ST48 tet(X4)-harboring isolates closely related strains

**Fig 7:**
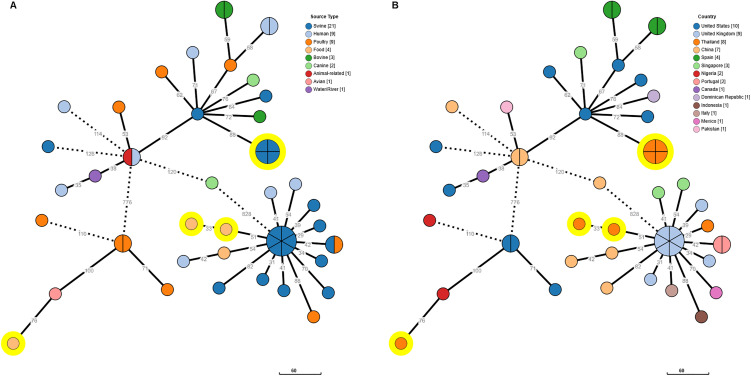
A minimum spanning tree based on core genome MLST (cgMLST) analysis of ST48 *tet*(X4)-harboring isolates genetically related strains. The tree visualizes the relationships between ST48 *tet*(X4)-harboring isolates from this study (highlighted with yellow) and closely related strains (HierCC 100) retrieved from Enterobase. The trees are annotated by type of sample (A) and by country of origin (B), to reveal the global distribution and epidemiological context of these related strains. Nodes highlighted in yellow represent isolates from the current study. Node sectors are proportional to the number of isolates, and branches lengths indicate the allelic difference between nodes.

A total of 44 closely related strains to ST48 *tet*(X4)-harboring isolates were identified in public databases, defined by a HierCC 100 (Fig 7, S5 Table). The sources of these strains revealed potential inter-sectoral dissemination. The most frequent source was swine (17 strains), followed by human and poultry (9 strains). Bovine, canine, and environmental strains from river water were less commonly found (Fig 7A). Interestingly, the geographical distribution of these related strains was mainly found in the United States and the United Kingdom (Fig 7B). For Asia country, most of the strains originated from China and Singapore. Only one strain isolated from swine in Thailand was found to be closely related to our isolates. These demonstrate the international dissemination potential of these strains.

## Discussion

The circulation of AMR bacteria in the food chain significantly increases food safety risks for consumers and can negatively impact meat product exports. Our findings demonstrated that the ESBL-producing *E. coli* isolates from retail market pork carried diverse ARG profiles that overlap with cecum samples from the slaughterhouse located in Suphan Buri, suggesting a transmission link between these two sources. This inter-source spreading of ARGs is consistent with previous source attribution studied in pork and chicken production chains, confirming that slaughterhouses were the original source of ARGs in *E. coli* isolates [22]. The diverse ARG profiles further indicate the complexity of the contamination, suggesting multiple potential sources within the processing and retail environment, such as butchers, equipment (e.g., cutting boards, knives, meat grinders), and the ambient market surroundings. The multi-source contamination and the dissemination of diverse ARGs are supported by several studies assessing the prevalence and variety of ARGs present in meat products [23]. Additionally, a significant number of shared ARGs between pig and human isolates was reported, underscoring the critical role of pork production in AMR [24].

Our ESBL isolates exhibited a high prevalence of co-harboring multiple ARGs, most notably *floR, tet*(A)*, qnrS1,* and *aac(3)-IId*, conferring resistance to phenicols, tetracyclines, quinolones, and aminoglycosides, respectively. These findings align with the reports of ESBL-producing *E. coli* isolates from minced meat at Thai local markets, which demonstrated MDR phenotypes against critically important antibiotics [25]. The high frequency of these ARGs likely reflects the selective pressure from antibiotic classes historically and currently utilized in Southeast Asian pork production [4]. Furthermore, detection of *bla*_OXA-1_, *bla*_NDM-5,_ and *mcr-1.1* in our isolates highlights resistance to the critically important antibiotics carbapenems and colistin. This occurrence indicates the global emergence of last-resort resistance in retail meat, as observed in South Africa [26]. Crucially, *tet*(X4), conferring resistance to the last-resort antibiotic tigecycline, was identified. Large-scale database curation has highlighted the distribution of *tet*(X4)-producing *E. coli* within Southeast Asian countries was comparatively low in public repositories. However, Thailand has exhibited a higher number of *tet*(X4) (n = 28) compared to Vietnam (n = 9), Malaysia (n = 7), Singapore (n = 4), and Cambodia (n = 3) [27]. Although AST for tigecycline was not included in our initial screening panel, the identification of the *tet*(X4) via whole-genome sequencing provides a highly reliable predictive framework for the resistance phenotype. It is well established that the presence of this gene is strongly associated with high MIC and resistance to tigecycline [28,29]. These findings collectively highlight the critical importance of antibiotic resistance hotspots in meat products, posing a potential threat to public health.

Virulence factors (VF)-encoding gene profiling indicates pathogenic potential and relatedness between isolates by virulence traits. Consistent with the resistome, our findings demonstrated shared VF gene profiles between isolates from retail pork markets and the slaughterhouse, supporting molecular evidence of genetic relatedness. The common VF genes found in our study, including *csgA, terC, gad,* and *nlpI*, are also observed in pork, slaughterhouses, and farms *E. coli* isolates from Portugal and South Africa [30,31]. Additionally, most of our isolates possessed VF genes associated with ExPEC, underscoring a spreading of highly pathogenic strains in the pork supply chain. Previous research further supports the clinical relevance of pork isolates by reporting a potential association between pork and patients *E. coli* strains by identifying a similar set of VF genes found sharing a similar set of VF genes [32]. Furthermore, our multivariate analysis reveals a distinctly divergent behavior between the resistome and virulome of *E. coli* isolates as they transition along the pork production chain. While the resistome is actively influenced by the sample source, the virulome remains completely uniform across both sources. These contrasting dynamics demonstrate that while processing may introduce localized selective filters or open ecological niches for the horizontal gene transfer of resistance genes, the underlying pathogenic machinery remains entirely intact and uniformly distributed from slaughterhouse to markets. Crucially, these suggested that the conservation of these VF genes might facilitate bacterial persistence along pork production.

AMR dissemination possibly occurs *via* MGEs or clonal spreading, both enabling bacteria to rapidly acquire and share ARGs across different niches. In this study, the mobilome co-contig with ARGs and VF genes was highly diverse, indicating the high potential for transferability of multiple ARGs in a food chain. Specifically, we investigated that the *bla*_CTX-M-14_ and *qnrS1* were associated with IS*Ec9*, IS*102*, IS*26*, IS*Kpn19*, while *bla*_CTX-M-55_ was found in proximity to IS*Ec9*. This finding is aligned with many reports where genes encoding ESBLs were also flanked by IS*Ec9* [33–35]. Although the IncF type was identified as the most common plasmid replicon type across all our isolates, IncQ, IncR, IncX, and IncY also demonstrated an association with ARGs. This observed diverse plasmid replicon profile is consistent with regional findings, IncF_repB_ and IncY were commonly identified across humans, pigs, pig carcasses, and pork in Thailand and Lao PDR [36]. While IncX carrying *qnrS1* was the most frequently isolated from commensal *E. coli* from the pork and beef production chain in Germany [37]. Additionally, we found that both IncF and IS*26* were mainly associated with the specific VF genes (*anr* and *traJ*). This finding is consistent with the study, which demonstrated that a set of VF genes (*traT*, *ompT*, *etsC*, *hlyF*, *anr*, and *traJ*) exhibited a strong inferred link with the IncF plasmid type in retail meat isolates, although most VF genes were not directly linked to IS or transposons [38]. Collectively, this evidence confirms that the HGT mediated by this diverse mobilome is the primary genetic mechanism facilitating the circulation and complexity of AMR in the pork production chain.

The population structure analysis revealed that phylogroups A and B1 were most prevalent, consistent with other studies [30,32]. The dominant ST48 clone, belonging to phylogroup A, was highly prevalent in both pork and cecum samples and widely disseminated across several Bangkok retail markets in different districts, implying potential cross-contamination or originating from an upstream source. Notably, this lineage was the primary carrier of the *tet*(X4) gene, further establishing it as a high-risk vehicle for tigecycline resistance. In contrast, a study in China has reported the clonal spread of ST195 *tet*(X4)-producing *E. coli* in retail vegetables, which is closely related to strains from pigs, pork, and humans across regions [11]. Other shared STs (ST10 and ST201) found in pork from different markets also indicate the possible widespread dissemination of these clones. Similarly, ST10 and ST48 were commonly identified among isolates from pig farms, slaughterhouses, and terminal markets in Henan Province in China and Portugal, suggesting that these clones may originate from pig farms and subsequently disseminate across the entire production chain to the markets [30,39]. Crucially, the significant SNP differences observed between ST48 pork and cecum isolates suggests independent strain acquisition and shared evolutionary ancestry among these *E. coli* lineages, rather than direct clonal spread along the supply chain. The accumulation of these SNPs indicates evolutionary divergence that typically requires days or weeks and is unlikely to occur through persistence in the food processing chain [40]. However, the remarkably conserved local genetic context of *tet*(X4) observed across both sources demonstrates that the dissemination of this resistance determinant is driven by highly active HGT events. Notably, the high-risk ST48 *tet*(X4)-positive isolates from cecum samples demonstrated low SNP differences, indicating a pattern of localized clonal expansion within the slaughterhouse. Together, these findings demonstrate that while this high-risk ST48 lineage utilizes clonal expansion to persist locally, it relies on horizontal gene transfer to distribute resistance genes across the broader pork production chain.

We identified a conserved *estT*-*tet*(X4)-IS*CR2* putatively active mobile genetic unit in ST48 isolates carrying *tet*(X4), structurally comparable to genetic environments reported in previous studies [41–45]. In addition, the flanking IS*CR2* element is capable of mediating the rapid shuffling of *tet*(X4) into more stably maintained environments, such as the bacterial chromosome [41,46]. The conservation of this specific flanking architecture strongly supports the likelihood that HGT facilitates the broader dissemination of *tet*(X4) from the slaughterhouse to retail pork meat. Similarly, other critical ARGs, such as *bla*_NDM-5_ and *mcr-1*, have been found to disseminate via their specific conserved active mobile units. The composite transposon Tn*125* (ΔIS*Aba125*-IS*5*-*bla*_NDM-5_-*ble*_MBL_-*trpF*-*dsbD*) and the *mcr-1*-*pap2* cassette were conservatively identified, which may further facilitate persistence of these resistance genes in the food-chain ecosystems [47]. The cgMLST provided an assessment of the genetic relatedness of our *tet*(X4)-positive ST48 isolates with global strains. They were genetically closely related to strains from diverse hosts, including humans, animals, and the environment in various countries. This close genetic relationship underscores a One Health concern, suggesting that these high-risk isolates are circulating across different hosts or environments. The presence of nearly identical core genomes in both porcine and human-derived strains implies either a direct zoonotic transmission event or a shared exposure to a common contaminated source within the regional ecosystem.

The primary limitation of this study is the relatively small number of final sequenced isolates, which may restrict the generalizability of our findings across distant geographic regions and varied production systems. However, we utilized a targeted β-lactamase gene screening framework to ensure that this sequenced cohort effectively represents the structural genetic diversity of the broader circulating *E. coli* populations within these specific settings. Additionally, the reliance on short-read sequencing technology presents an inherent technical constraint. While our short-read data clearly resolves the immediate genetic flanking environment, determining the complete global plasmid backbones requires future long-read validation. Future large-scale studies incorporating long-read sequencing and conjugation assays are warranted to definitively track the long-term evolutionary dynamics and horizontal transfer potential of these high-risk clones across diverse production chains and environmental reservoirs.

This study has provided crucial insight into the transmission dynamics that define the AMR risk in the central Thailand pork supply chain. The ESBL-producing *E. coli* populations in pig cecum at the slaughterhouse and pork meat at the retail market shared an evolutionary ancestor. The dissemination of ARGs across these two sources is mediated mainly through HGT, rather than direct clonal spread. Especially, the identification of high-risk *tet*(X4)-positive ST48 isolates harboring the conserved *estT*-*tet*(X4)-IS*CR2* transposition unit underscores the rapid mobility of the last-resort resistance gene. Therefore, the Hazard Analysis Critical Control Point (HACCP) system and hygiene practices throughout the pork production chain are suggested to reduce the potential transmission of AMR *E. coli* to the consumer.

## Supporting information

S1 Fig Chromosomal point mutations responsible for fluoroquinolone resistance. A heat map illustrating the presence of chromosomal point mutations across isolates.
(TIF)

S2 Fig Co-contigs of Inc types and IS with ARGs and VF genes. (A) Co-contigs of major Inc type and IS with specific ARGs. (B) Co-contigs of major Inc type and IS with specific VF genes. Each icon represents an observed co-contig event, and the size of the icon corresponds to the number of co-contigs of that specific Inc-ARGs, IS-AGRs, Inc-VF genes, and IS-VF genes observed across each source.
(TIF)

S1 Table AST panel of the analyzed *E. coli* isolates from pig cecum (n = 14) and retail market pork (n = 26). (XLSX)

S2 Table Metadata and assembly QC metrics of the isolates. (XLSX)

S3 Table Number of Inc-ARGs co-contigs identified in *E. coli* isolates from both cecum and pork sources. (XLSX)

S4 Table Number of IS-ARGs co-contigs identified in *E. coli* isolates from both cecum and pork sources. (XLSX)

S5 Table Metadata of the closely related strains (HierCC 100) retrieved from Enterobase. (XLSX)
